# Older patients’ participation in team meetings—A phenomenological study from the nurses’ perspective

**DOI:** 10.3402/qhw.v8i0.21908

**Published:** 2013-12-20

**Authors:** Elisabeth Lindberg, Eva Persson, Ulrica Hörberg, Margaretha Ekebergh

**Affiliations:** 1 School of Health Sciences, University of Borås, Borås, Sweden; 2 School of Health and Caring Sciences, Linnæus University, Växjö, Sweden; 3 Board of Health Sciences, Faculty of Medicine, Lund University, Lund, Sweden

**Keywords:** Healthcare professionals, older people, patient participation, phenomenology, team meeting, ward round

## Abstract

Although the importance of patient participation is acknowledged in today’s healthcare, many challenges remain before patient participation can become an integral part of care provision. The ward round has traditionally been the forum for crucial decisions about patient care, but often with limited possibilities for patient participation. As part of the process of improving patient participation, the round in the present study has been replaced by a team meeting (TM) to which the patient has been invited. The aim of this study is to highlight nurses’ experiences of older patients’ participation in TMs. The research process was guided by the principles of phenomenological reflective life world research. Data were collected in a Swedish hospital, in a ward specializing in older patients. Nine nurses, who had invited and planned for a patient to participate in TMs and/or had experienced TMs in which patients participated, were interviewed. The essential meaning of patient participation in the TM, as experienced by the nurses, is that patient participation can be supported by a safe relationship in which the patient can make his or her voice heard. Participation is challenged by the patients’ vulnerability and by the subordinated role assigned to the patient. The essential meaning is further described by its constituents: “the need for a guide,” “patient participation challenged by structures,” and “creating space for the whole human being.” In conclusion, the nurse plays a core role in guiding the patient in an unfamiliar situation. The meaning of patient participation in the TM needs to be discussed by professionals so that the patient perspective is present.

The present article is part of a research project which focuses on the phenomenon of older patients’ participation in team meetings (TMs). In an earlier study within the present project, the patient’s experience of participating in a TM is described (Lindberg, Hörberg, Persson, & Ekebergh, 2013). The results from that study indicated a need to supplement the research with the nurses’ experiences of patient participation in a TM, which is thus the focus of this article. Nine nurses, who had invited and planned for a patient to participate in TMs and/or experienced a TM in which a patient was invited, were interviewed.

Patient participation, especially participation in determining treatment and care, is perceived as a right in many Western societies (Eldh, Ekman, & Ehnfors, 2006), but a clear understanding of the concept of patient participation remains elusive and complex (Sahlsten, Larsson, Sjöström, & Plos, 2008). Patient participation has been described as an established relationship between the nurse and patient in the context of nursing practice. The nurse, for example, must surrender some power or control to and share information and knowledge with the patient (Sahlsten et al., 2008). For patients, the experience of participation in their own care makes them feel less vulnerable and more able to maintain a sense of control in the situation (Eldh, Ehnfors, & Ekman, 2006; Frank, Asp, & Dahlberg, 2009). Lyttle and Ryan (2010) showed in the context of older people that there is a need for care that allows older patients to influence their own conditions based on their own desires. Patients can participate in their own care even if they are old and frail (Tutton, 2005). At the same time, a caring relationship is vital if older patients are to experience that they are actually participating (Andersson, Burman, & Skär, 2011; Bridges, Flatley, & Meyer, 2009; Jonasson & Berterö, 2012; Tutton, 2005). Patients have different needs for participation, and it is important that the individual patient is the starting point for all care actions (Frank et al., 2009). This emphasizes the importance of taking a lifeworld perspective in which the concept of participation is grounded in a patient perspective (Dahlberg & Segesten, 2010). Participation can be promoted by taking time to listen to the patient’s story, and, as Dahlberg, Todres, and Galvin (2009), p. 270) suggest, a shift from patients as “consumers” to patients as “storied beings” needs to take place.

The TM is in the present article to be understood as a replacement for the ward round. The latter is a traditional forum for discussing patients’ care and medical treatment. The traditional round, with its strong focus on biomedical science, has in recent years come to be questioned, in favour of a more integrated approach in which different kinds of knowledge are valued equally and in which the patient perspective is essential (Fiddler et al., 2010; Manias & Street, 2001; Sweet & Wilson, 2011). Research also indicates that interactions during the round often follow a specific pattern in which the physician poses questions to the patient, focusing on medical issues. Nurses participate to a more limited extent than both patients and physicians, and tend not to raise questions other than those of a purely medical nature (Weber, Stöckli, Nübling, & Langewitz, 2007). This limited involvement is in contrast to research indicating that nurses’ active participation in ward rounds has been found to be of importance for patient safety and increased quality of care (Desai, Caldwell, & Herring, 2011; Parissopoulos, Timmins, & Daly, 2013). It may also be difficult for patients to get involved in the discussion. Patients are at times ignored when they try to describe feelings connected to the experience of illness, causing them to feel both ignored and neglected (Sweet & Wilson, 2011). A TM can be one way of developing the ward round, but patient participation in TMs is described as difficult to achieve (Opie, 1998; Parker Oliver, Porock, Demiris, & Courtney, 2005; Vuokila-Oikkonen, Janhonen, & Nikkonen, 2002). The trustful and supportive relationship that the patients describe as being important for their possibilities of taking part in TMs sometimes seems difficult to establish, and instead the patients experience the professionals as “shy and silent” and with a limited interest in their life situation (Lindberg et al., 2013).

As indicated in earlier research, patient participation is a complex phenomenon, especially when participation is to be achieved in situations traditionally carried out with little involvement from the patient, such as in a ward round or a TM. The results from the earlier study within our research project indicate that the patients needed support from the professionals in order to achieve participation. The results also indicate that the patients’ vulnerability due to illness and an ageing body posed challenges in the process in which they became participative and that the patient wanted to be confirmed as a whole human being when participating in a TM (Lindberg et al., 2013). Nurses, as part of the healthcare team, have an important role in creating opportunities for patient participation, but as indicated earlier in this article, it can be difficult for nurses to have space in situations such as a TM or a ward round. One way to develop the TM so that the patient becomes more involved and at the same time is given the opportunity to become prominent as a whole human being could be by reinforcing the patient perspective with support from the nurse. Based on this, together with a need for a broader perspective on patient participation in TMs, it is important to gain knowledge about the nurse’s experiences of older patients’ participation in the TM. The overarching research question in this study is thus: What constitutes older patients’ participation in the TM from the nurses’ perspective? The aim of the present study is to highlight nurses’ experiences of older patients’ participation in TMs.

## Method

The study is descriptive in design and based on a theoretical framework grounded in lifeworld phenomenology and caring science. This can be seen in this study through the search for the nurses’ lived experience of patients’ participation in a TM, where participation is based on a holistic patient perspective.

The research process was guided by the phenomenological approach of reflective lifeworld research (RLR) (Dahlberg, Dahlberg, & Nyström, 2008). This approach is based on the phenomenological philosophy of Husserl (1936/1970, 1929/1977) and Merleau-Ponty (1964/1968, 1945/2011). The goal of RLR is to describe human beings’ lived experiences of a phenomenon so that a greater understanding of the phenomenon is achieved. In RLR (and phenomenology), the concept of the phenomenon is important. A phenomenon can be understood as an object or a matter as it presents itself or as it is experienced by a subject (Dahlberg et al., 2008, p. 33). A phenomenon reveals its deeper significance through a continuous search for meaning and through rich descriptions from the informants. This process allows the phenomenon to appear as clear and understandable (Dahlberg et al., 2008).

RLR requires openness and flexibility towards the phenomenon that is studied, which in this study is older patients’ participation in TMs as experienced by nurses. In order to retain the openness as long as possible and to enable what is not directly visible to become visible, it is important that the whole of the research process is characterized by a reflective attitude. This reflective attitude is described by Dahlberg et al. (2008) as bridling. The act of bridling can, according to the authors, be seen as an art, in which thoughtfulness, openness, reflection, and close examination are important components in the search for the meaning of the phenomenon. RLR requires openness to the Lifeworld throughout the process, and this includes openness to oneself as a researcher (Dahlberg et al., 2008). Throughout the research process, reviews and discussions have taken place which have helped in highlighting hidden aspects, noting the present authors’ pre-understandings.

In the phenomenological tradition, an understanding of other human beings’ experiences is possible through Husserl’s (1929/1977) and Merleau-Ponty’s (1945/2011) descriptions of intersubjectivity. Both Husserl and Merleau-Ponty describe intersubjectivity as pertaining to a world that is individual but at the same time shared. In our common world, our understanding of other human beings is grounded in our shared experience as humans: We cannot experience the same as the other, but we can experience that the other is an experiencing subject. The nurses’ experiences of older patients’ participation in the TM is facilitated through intersubjectivity and through phenomenology where focus on “the things themselves” (patients’ participation in TM), how “the things” appear to somebody (the nurses), and how they are made explicit through language and conceptualization (in the research process) creates the foundation.

## Setting and participants

The study was conducted in a ward for people >75 years of age, at a hospital in western Sweden. All patients were living in ordinary housing, although a majority of them needed some kind of home care service or support from friends and relatives to manage their daily lives. The reasons for hospitalization differed, but a fairly common cause was multiple medical diagnoses in combination with difficulties in managing everyday life at home. The ward had 15 beds, and the planned hospitalization was ideally not to exceed 3 days.

The traditional ward round had been developed into a TM in the ward where the study took place, and representatives from various professions gathered to discuss patients’ treatment and care. The TM had the form of a seated meeting which took place every weekday. After some time, several professionals drew attention to the lack of a patient perspective during the TM, and consequently a decision was made to invite patients to participate.

At the time of the study, 12 nurses were employed. The criterion for inclusion was that the nurse had invited and planned for a patient to participate in TMs and/or had experienced patient participation in TMs. Nine nurses (all female) had this experience and were therefore invited, via an information letter and a follow-up call, to participate in the study (eight nurses had experienced patient participation in the TM and one nurse had invited and planned for a patient to participate, but due to illness this patient was not able to participate on the day for the TM). All of these nurses accepted the invitation. At the time of the interviews, the nurses were between 25 and 45 years of age (mean age 35.4) and had worked as nurses between 1 and 25 years.

## Data collection

Life world interviews (Dahlberg et al., 2008) took place in a conference room at the hospital and lasted between 40 and 65 min. They were recorded and subsequently transcribed verbatim. An attitude of adherence and openness towards the phenomenon and a bridling of the researcher’s pre-understanding guided the interview. The participants were initially asked to describe a situation in which a patient had been invited to participate and/or participated in a TM. Follow-up questions, for example “How is that …?” or “Could you tell me more about …?”, were then posed in order to gain a deeper understanding of the phenomenon of interest.

## Data analysis

The data were analysed according to the principles of RLR, as described by Dahlberg et al. (2008). The principles acted as a “guiding light” in the process of analysis, and the different parts can be seen as parts in an intertwining process rather than as fixed steps. After transcribing the interviews (which were performed by the first author), the analysis started by reading the text several times as a whole in an attempt to obtain an overall sense of the material. The text was then divided, according to the meanings that were observed, into meaning units. Unpacking the meaning units was then performed with a bridling approach (Dahlberg et al., 2008). This mainly entailed asking questions of the text, such as: How does patient participation during the TM appear to the nurses? Which meaning (in relation to the phenomenon) can be seen in this part of the text? Meanings were brought to the meaning units through reflection and questioning. In the next phase, clusters of meaning were created to see the essential meanings. A cluster is a preliminary structure of meanings that is created based on differences and similarities in meanings. By relating the clusters to each other, the clusters support the search for the meaning structure (the essence) of the phenomenon. The essence is the most abstract level of the analysis (Dahlberg, 2006; Giorgi, 1997), and in the present study the essence was formulated by a questioning and reflective attitude, where questions such as the following were put to the text: What is the meaning of patient participation from the nurses’ perspective? How does participation in the TM appear from the nurses’ perspective? The essence emerged through a movement between the parts and the whole. Once the essence had been formulated, the analysis continued by describing the more contextual nuances of the phenomenon, in RLR named as “constituents” (Dahlberg et al., 2008).

## Ethical considerations

The present study was conducted in a relationship with healthcare staff working in one unit. It was therefore necessary that both participants and the situation be treated with respect; ethical considerations were thus given a heightened focus throughout the research process.

The study was approved by the Regional Ethical Review Board of Gothenburg (No. 757-09). The study was carried out in accordance with the principles outlined in the Declaration of Helsinki (World Medical Association, 2008). The participants were informed both orally and in writing that participation was voluntary and that confidentiality would be maintained. Written informed consent was obtained from each participant.

## Results

The essence of the phenomenon will be presented first, followed by its three constituents. The essential meaning of older patient participation in the TM, as described from the perspective of the nurses, is that the TM is constantly present in care provision and is not limited in time and space. The time before, during, and after the TM is intertwined into a whole in which patient participation requires attention.

Patient participation is maintained when the patient receives and is encouraged to take space as a whole human being, is listened to, and can make his or her voice heard. Patients’ participation is also strengthened by safe, caring relationships in which the patient can be guided in the situation. Participation is challenged by the patients’ vulnerability and by the subordinated role assigned to the patient. The patient is easily reduced to an object if he or she is not able to follow the agenda of the TM’s unwritten rules. Patient participation also affects the relationship between the professionals of the TM, and the patient perspective is challenged by the professionals’ need for maintaining familiar patterns. Striving for such a familiar structure maintains patterns that promote the professionals’ security and safety at the expense of the patient.

The following constituents further elucidate the meaning of the phenomenon: the need for a guide, patient participation challenged by structures, and creating space for the whole human being.

## The need for a guide

Based on the nurses’ experiences, the periods before, during, and after the TM are intertwined and constantly present in care provision, independent of both time and space. The nurses describe how important it is that the patient has access to a guide who can help the patients find their way and avoid getting lost in the stream of events and experiences that characterize being admitted to hospital.

The nurses experience the TM as an unknown situation for the patient. In order to be able to create conditions for patient participation, the nurses describe the importance of preparing the patient before the meeting and that they work to create a safe relationship in which they, together with the patient, can prepare the latter for participation in a TM.

I prepared the patient over the weekend so that on Monday he/she was able to take part in the TM, and then it’s important that you have thought through whether you have any questions and so on.

The patients’ need for a guide continues during the TM. According to the nurses, they are able to support patients in such a way that participation is encouraged, based on their previous relationship. The patient may, for example, be supported by getting his or her personal history clarified by the nurse, which is described as being important for the patient. The patient’s personal history would otherwise have been ignored without recognition. The nurses describe how the patients can be “forgotten” during a TM; the nurse may then encourage participation by bringing the patient into the conversation: “they [the professionals] wanted it to be as usual, going quickly through each case and then moving on. I felt I had to go in and help her.”

The patient’s possibility of receiving guidance and support is influenced by the intensity of the TM. This means that the patient becomes subordinated when a nurse feels forced to give priority to and focus her attention on certain matters. The nurses describe how this need to establish priorities means that the function as a guide had to be set aside in certain situations.

And then unfortunately, or from my side anyway … I feel that the patients get left behind. There are all these forms … and which tests are to be carried out on the patients.

The patient in many cases needs further information after the TM, and the nurses experience that it is important that they provide support by being able to answer questions. As the TM is not limited in time or space, the nurses describe how the patients need them as guides throughout their time at the hospital.

The patient’s participation in the TM does not necessarily entail that he or she is physically present during the meeting. The fact that the patient can be vulnerable means that he or she does not always have the strength to attend the meeting, and in some situations the nurse experience that she must make decisions about whether or not a patient should be present. As exemplified in the two following descriptions, either the nurse can choose to represent the patient during the TM or she can choose to bring the patient to the TM despite the fact that the patient is in a vulnerable position. In the first example, the nurse’s guiding function involves her representing the patient during the TM. One way in which the nurse can do this is by listening to the patient’s personal history or observing him or her. In this way, the nurse creates the opportunity to gather information that allows her to decide in which way the patients’ interests best can be represented during the TM.

But then in the morning I knew she had really changed, she had difficulty standing and wobbled and was very confused when talking. And then I felt: “Either I take her with me and the TM will be of poor quality… or I leave her behind and try to speak for her at the meeting.”

In the second example, the nurse takes a patient to the TM despite the fact that the latter’s health had deteriorated during the night. At the TM, the patient’s deteriorated health is obvious to the professionals, and as soon as that fact has been established, some of the professionals lose their interest in the patient’s narrative and begin to discuss among themselves.

Everybody realized that we had to X-ray her brain … but the patient herself didn’t understand this.… The doctors … talked a little with each other, whereas the patient sat and repeated her story.

In this situation, the nurse experiences the patient as being excluded and omitted without any guidance and support from the professionals.

## Patient participation challenged by structures

The TM is a routine procedure for the professionals, according to the nurses, in that everyone knows what is expected of them. For the patient, however, the environment and the situation are unfamiliar. When the professionals fail to see the patient’s vulnerability, the nurse experiences the patient as being isolated. One such situation can arise in terms of how the professionals position themselves in relation to the patient. A nurse describes how she and the physician, by positioning themselves next to each other, form a team that confronts (instead of supports) the patient: “I sat opposite the patient and the doctor sat next to me; it felt a little like the doctor and I were a team while we confronted her.” The patient perspective can in these situations be obscured, and as described by the nurses it can be difficult for patients to demand attention and ask questions. In such cases, participation is reduced to mere presence: “She didn’t say very much on her own: it was more the doctor who told her what we had seen.”

Based on the nurses’ experiences, the patients are not always given proper support to achieve participation. This may occur, for example, when a patient’s wishes fall outside the rules governing how social interventions can be offered to older people. One nurse gave an example of a meeting with an older woman who wanted to be moved to a nursing home: “She couldn’t manage to go home to her big house, it was overwhelming for her. The fact that she was ill was enough! But she wasn’t ill enough!” The vulnerability of this woman touched the nurse and contributed to a sense of resignation, owing to the lack of respect for the patient’s voice, “but I felt she was overruled in this. And she didn’t have much time left, so it’s a shame that she was not able to decide how she wanted things to be arranged.”

According to the nurses, the possibility of the patient participating is affected by how the professionals relate to each other and how they relate to patient participation in the TM. One nurse describes how she and the nurse assistant had prepared a number of patients for participation in the TM, but when the physician arrived, he or she did not want the patients to participate. Even if there was a joint decision in the professional group to invite patients, this was not realized in the current situation, meaning that the commitment to allow participation for the patients was broken, which was also recognized by the patients, as one of them later asked the nurse, “Wasn’t I supposed to attend some meeting?”.

Patient participation can be affected by choices made by the professionals. In some situations (as, e.g., in the situation above), the patient can be rejected; in other situations, where a patient perspective is more evidently adopted, the interests of the patient are protected. A nurse describes how she stood by her invitation, but was then forced to accept the displeasure when the TM went on too long: “I felt the doctors were irritated that day … it was probably directed to me as a nurse.” Even in the face of time pressure and irritation on the part of other professionals, the nurse described the patient’s participation as valuable.

## Creating space for the whole human being

According to the nurses, patients’ vulnerability and the existential dimensions of the caring relationship are brought to the fore in many cases: “she was talking a lot about her family … that the children didn’t have time any longer and that she was sad.” Patients need to talk about how they experience their life. Just as the TM can be present in care provision, care provision can also be present during the TM.

maybe it’s part of what they want support in … so when they enter into a context where there are a lot of people listening to just them, well, maybe they take the chance to talk for a while. But then at least this has been achieved!

The patients are, according to the nurses, affected by an organization under pressure from time constraints. The patients need to be listened to, and they also need opportunities to tell their personal histories which require that the nurse sets aside the demands for efficiency placed on her. Instead of being listened to, the nurses describe how the patients are caught up in a situation where the professionals set the agenda for what can be spoken of during the TM: “Now as soon as we’ve seen what we need to see, then it feels like time is running out and we have to move on to the other patients.” Nurses describe how a mood of frustration is generated in the room when a patient tells a long story. For example, professionals may look at their watches, browse through folders, or talk to each other. Sometimes, the patients are interrupted, not allowed to finish what they want to say: “the doctor, well, as I said he cut her off.” A nurse who realizes that a sense of frustration has begun to emerge due to time pressure does not always work to create space for the patient to participate, “to interrupt, maybe cut in on the doctor.… It’s just not done!” The nurses describe how their focus in these situations is moved from the patient towards a focus on trying to maintain the framework of the TM. In this situation, it is difficult for the nurses to be able to relax in the present encounter, and allow the patient to make his or her voice heard.

Patient participation in the TM is in many ways a challenge for the patient. Vulnerability due to disease and an ageing body affects the possibilities for dealing with participating. In some situations, the nurses describe the challenges as exceptional; this occurs, for example, when the patient has a diagnosis of dementia. The relationship between the professionals and the patient becomes important again, and the nurse describes how she can choose not to invite these patients to the TM, or she can choose to invite the patient and, based on the patient’s unique situation, try to create opportunities for participation: “But I think you should give the person a chance unless you think it will be too confusing for the patient.”

## Discussion

The present study addresses several challenges posed by increased patient participation, especially in the context of older people. This has also been described in earlier studies. For example, older patients in need of hospital care may experience that information is withheld from them and that their concerns are not taken into account (Aasen, Kvangarsnes, & Heggen, 2012), while other studies have revealed how older patients may perceive that the healthcare service staff have power over them (Ekdahl, Andersson, & Friedrichsen, 2010). The nurses participating in the present study expressed a desire to further develop a patient perspective. A TM, however, does not always present a straightforward opportunity. TMs may fail to achieve this particular objective, which is to create the conditions required for patient participation and to promote such participation. Nurses often find themselves in situations where they must argue *for* the patient and *against* attitudes embedded in the current structure.

The present study shows that nurses can have a core role to play in patients’ ability to get involved in care provision. The nurses describe situations in which they can choose between supporting and hindering participation. Making such a choice involves taking a risk, and nurses need to be courageous in order to facilitate patients’ participation. As suggested by Thorup, Rundqvist, Roberts, and Delmar (2012), courage is about taking a stand for the patient and acting in accordance with ethical values even in situations where there is a risk of being abandoned by colleagues. The results presented in this article contain descriptions of courage, acted out in situations where nurses found themselves alone, arguing for their patients and challenging conflicting attitudes about patient participation.

Nurses also described situations in which the nurse chooses to stand back and not give the patient the opportunity to participate. In some circumstances, nurses may thus fail to support a patient during a TM. Patients expect nurses to stand up for them, and they develop feelings of mistrust when a nurse fails to meet this expectation (Andersson et al., 2011; Larsson, Sahlsten, Segesten, & Plos, 2011; Lindberg et al., 2013). Conflicts among the healthcare team as well as stress arising from time pressure and workplace attitudes have been described as factors that reduce nurses’ ability to support their patients (Huntington et al., 2011; Lindberg, Persson, & Bondas, 2012).

The nurses’ descriptions include examples of how they try to create a secure atmosphere for the patient, and examples in which the vulnerability and exposure of the patient have remained. It has been found that a feeling of security, expressed by the older patients as “feeling at home,” is important for the outcome of their participation in a TM (Lindberg et al., 2013). However, Andersson, Hallberg, and Edberg (2008) show how a feeling of “not being at home” during a hospital stay can lead to feelings of loneliness and reduced well-being among older patients. According to the authors, these feelings are connected to the experience of being left out. The nurses’ descriptions in the present study contained glimpses of the patients’ longing and desire to tell their own story. Patients sometimes expressed a desire for security and comfort through these personal histories, only to be offered a practical (often temporary) solution. Illness causes a disruption in life and threatens to alienate the lifeworld. Svenaeus (2011) expands Heidegger’s concept of “homelessness” (“unheimlichkeit”) (Heidegger, 1962) and describes how illness creates cracks in life, allowing the past and present to appear in a sense of alienation or homelessness. One way of making sense of this alienation and creating meaning is for people to be able to recount their personal histories. Dahlberg et al. (2009) show how stories can support well-being, and, as described by Lindberg et al. (2013), the opportunity to tell one’s story during the TM is of vital importance in the process of creating meaning in an alien environment. A TM that is a place where stories can be told has a deeper meaning than a TM in which professionals talk about patients. It is thus possible for a TM to become an integral part of care provision where participation is created in the relationships, based on the patient’s story.

Entering a room in which a TM is taking place may make a patient feel vulnerable. The nurses’ descriptions include examples of how they have succeeded in creating a secure atmosphere for the patient, and examples in which the vulnerability and exposure of the patient have remained. Svenaeus (2011) believes that it is the responsibility of professionals to understand the unhomelike feeling of “being-in-the-world” and to support the patient, so that he or she can regain a feeling of “homelikeness.” A TM is not limited in time and space, and a nurse who acknowledges this can guide a patient in this situation. A nurse who prepares a patient for a TM is inviting the patient to find a role (and occupy space) in the room, while following up the patient after the TM can create the conditions required for a feeling of homelikeness.

## Methodological considerations

The TM can be understood as an institutional conversation, in which inherent traditional structures are more or less visible. The TM can also be interpreted as an intersubjective meeting in which humans come together and create a unique situation. In this study, intersubjectivity as described by Husserl (1929/1977) and Merleau-Ponty (1945/2011) creates a foundation for the nurses’ description of patient participation in the TM. Through their stories, the nurses contribute to an extra dimension and a deeper understanding of how organizational and hierarchical structures as well as individual choices made by the nurse can affect the patients’ possibility for participating.

All nurses included in this study, except one, had experienced patient participation in a TM. The nurse without such experience had invited a patient to participate, but rapid deterioration of the latter’s health had prevented participation. The interview with this nurse, however, contained material relevant to the phenomenon of interest, and the interview was included in the analysis. To include this interview was also a way of being open towards the phenomenon. As the results indicate, the TM is not limited in time or space, the efforts made by the nurse to invite the patient and her decision not to bring the patient to the TM is therefore of importance for the phenomenon.

We have tried to perform the study by using a “bridled” approach and focusing on the phenomenon. Reflections and discussions in the research group, as well as in seminars, have been one way to ensure the validity of the results. The decision to invite the patient to participate in the TM was conducted on one single hospital ward, which can limit the transferability of the results. However, the fact that development of the TM was carried out based on practical experiences may ensure relevance to clinical practice. Although the context of the study is a hospital ward for people >75 years of age, the results indicate traces of characteristics that can be universal for humans regardless of age. Further studies are needed that can elucidate different perspectives on older as well as younger patients’ participation in TMs or ward rounds. These studies can, for example, be interventional in nature and take into consideration the complexity of the concept of “participation” as well as include the voice of the patient.

## Conclusions

Inviting an older patient to a TM brings the question of participation to the fore. The choices made by the nurse affect both the patient and the nurse. The patients and nurses are intertwined in the caring relationship, where the nurse is responsible for the care and the patient is affected by the nurse’s ability to support him or her. When the patient was invited, the nurse was able to create conditions for participation, or she could simply choose to let the patient be present. Reflecting on the outcomes of choices made and learning from past situations can be two fruitful ways of strengthening and further developing the patient perspective.

The present results show how important it is that nurses “take heart” and explicitly clarify the value of a patient perspective. The nurses describe how the patients need them, not as a silent shadow hiding behind other professionals but as a brave guide who acknowledges patient’s own value and skills, and dares to walk unknown paths alongside the patient. Adopting a patient perspective is a common responsibility for all professionals involved in patient care, and the guiding function, which is in accordance with a caring science perspective, could then apply to all professionals involved in a TM.
